# Interdependence of clinical factors predicting cognition in children with tuberous sclerosis complex

**DOI:** 10.1007/s00415-016-8335-5

**Published:** 2016-11-22

**Authors:** I. E. Overwater, B. J. H. Verhaar, H. F. Lingsma, G. C. B. Bindels-de Heus, A. M. W. van den Ouweland, M. Nellist, L. W. ten Hoopen, Y. Elgersma, H. A. Moll, M. C. Y. de Wit

**Affiliations:** 1 Department of Neurology, Erasmus University Medical Centre, PO Box 2060, 3000 CB Rotterdam, The Netherlands; 2 Department of Public Health, Erasmus University Medical Center, PO Box 2060, 3000 CB Rotterdam, The Netherlands; 3 Department of Pediatrics, Erasmus University Medical Center, PO Box 2060, 3000 CB Rotterdam, The Netherlands; 4 Department of Clinical Genetics, Erasmus University Medical Center, PO Box 2060, 3000 CB Rotterdam, The Netherlands; 5 Department of Child and Adolescent Psychiatry, Erasmus University Medical Center, PO Box 2060, 3000 CB Rotterdam, The Netherlands; 6 Department of Neuroscience, Erasmus University Medical Center, PO Box 2060, 3000 CB Rotterdam, The Netherlands; 7 ENCORE Expertise Center for Neurodevelopmental Disorders, Erasmus University Medical Center, PO Box 2060, 3000 CB Rotterdam, The Netherlands

**Keywords:** Tuberous sclerosis complex, Intelligence, Intellectual disability, Epilepsy

## Abstract

Cognitive development in patients with tuberous sclerosis complex is highly variable. Predictors in the infant years would be valuable to counsel parents and to support development. The aim of this study was to confirm factors that have been reported to be independently correlated with cognitive development. 102 patients included in this study were treated at the ENCORE-TSC expertise center of the Erasmus Medical Center-Sophia Children’s Hospital. Data from the first 24 months of life were used, including details on epilepsy, motor development and mutation status. Outcome was defined as cognitive development (intellectual equivalent, IE) as measured using tests appropriate to the patients age and cognitive abilities (median age at testing 8.2 years, IQR 4.7–12.0). Univariable and multivariable regression analyses were used. In a univariable analysis, predictors of lower IE were: the presence of infantile spasms (*β* = −18.3, *p* = 0.000), a larger number of antiepileptic drugs used (*β* = −6.3, *p* = 0.000), vigabatrin not used as first drug (*β* = −14.6, *p* = 0.020), corticosteroid treatment (*β* = −33.2, *p* = 0.005), and a later age at which the child could walk independently (*β* = −2.1, *p* = 0.000). An older age at seizure onset predicted higher IE (*β* = 1.7, *p* = 0.000). In a multivariable analysis, only age at seizure onset was significantly correlated to IE (*β* = 1.2, *p* = 0.005), contributing to 28% of the variation in IE. In our cohort, age at seizure onset was the only variable that independently predicted IE. Factors predicting cognitive development could aid parents and physicians in finding the appropriate support and schooling for these patients.

**Electronic supplementary material:**

The online version of this article (doi:10.1007/s00415-016-8335-5) contains supplementary material, which is available to authorized users.

## Introduction

Tuberous sclerosis complex (TSC) is a genetic disorder, with a large variation in symptoms between patients. Up to 90% of patients suffer from epilepsy, often starting in infancy [2]. Two-thirds of patients have refractory seizures [2, 12]. Cognitive function is highly variable in patients with TSC. One-third of patients suffer from profound disability, while others have normal cognitive abilities [4, 8, 14].

Several studies have attempted to unravel the causes of this variation in cognitive development. Many studies have shown that epilepsy is associated with delayed development and impaired cognitive functioning [9]. In previous studies, refractory seizures and infantile spasms were correlated with lower cognitive functioning, while older age at seizure onset and early treatment correlated with better cognition [1, 5, 7, 8, 15]. Lesion burden on brain MRI has also been correlated with cognition, including the presence and number of cortical tubers [11, 13].

Most of these correlations were identified using a univariable analysis, between the factor of interest and the cognitive abilities of the patient. While it is useful to explore these correlations, it remains unclear what the actual contribution of a specific variable to cognitive functioning is, if the statistical method does not control for other possible influencing factors. This is further complicated by the rare nature of TSC; multivariable regression models can only hold multiple variables if sufficient patients are available to include in the analyses. In a previous study where a multivariable model was applied, the age at seizure onset was found to be the only independent predictor of IE [7].

In a cohort of 102 children with TSC, we aimed to confirm previously studied and identify new unstudied characteristics collected in the first 24 months of life, that are independent predictors for cognitive development later in life.

## Methods

Patients were selected from a retrospective follow-up clinical database at the ENCORE-TSC expertise center at the Erasmus Medical Center-Sophia Children’s Hospital. Missing data were completed through correspondence with physicians and other care providers. Of the total population of 121 children treated at our clinic, data on cognitive development were available for 102 children.

For all variables except intelligence equivalent (IE), data from the first 24 months of life were used. Mutation analysis was performed at the Department of Clinical Genetics of the Erasmus Medical Center according to standard procedures, using DNA extracted from peripheral blood. Success of the first AED was categorized as ‘yes’ if the first AED caused a seizure frequency decrease of 50% or more.

For all variables concerning epilepsy, children without epilepsy were given the most beneficial outcome, since previous studies have shown that the absence of epilepsy is beneficial for cognitive development [9]. The variables adjusted in patients without epilepsy are listed in Online Resource 1. In addition, some values were adjusted in children with epilepsy to include data from the first 24 months of life only; the variable ‘age at seizure onset’ was adjusted to 24 months (‘24’) in children with age at onset after 24 months of age (16 children), and for the variable ‘age of walking independently’, children who were not able to walk were assigned the value of 24 months (‘24’) (17 children).

The outcome of all analyses was IE, measured by the most reliable cognitive development testing based on the calendar age and cognitive abilities of the child. The Dutch versions of the following neuropsychological tests were used: Wechsler Intelligence Scale for Children (WISC; *n* = 45), Bayley Scales of Infant Development (BSID; *n* = 36), Wechsler Preschool and Primary Scale of Intelligence (WPPSI; *n* = 14), Snijders-Oomen Non-verbal Intelligence assessment (SON-R; *n* = 5), Attachment, Interaction, Mastery, Support (AIMS; *n* = 1) and Vineland Adaptive Behavior Scales (VABS; *n* = 1). Since some of these psychological tests measure development and not IQ, the value obtained from all tests was called intelligence equivalent, IE [6].

### Statistical analysis

For all continuous variables except IE, outliers were removed by only allowing data values within the 5th and 95th percentile, and adjusting values outside this range to the values of the 5th and 95th percentile. This adjusted three data points for the variable ‘age of waling independently’ and two for the ‘number of AEDs used’ variable. Multiple imputation was used to eliminate missing values. Missing values were present for the following variables: ‘number of AEDs used’ (6), ‘treatment success of first AED’ (7) and ‘age of walking independently’ (6).

IE values between children with and without epilepsy were compared using an independent samples *T* test.

Univariable and multivariable regression models were fitted with IE as outcome. Predictor variables in the regression model were selected from previous studies investigating the association between cognitive development and disease characteristics in patients with TSC. Variables with a Pearson correlation of >0.8 with other variables were left out of the model to prevent colinearity. This excluded the variable measuring the total number of months in which seizures were present during the first 24 months of life. Beta’s with 95% confidence intervals and corresponding p values were reported from the models. A variable was considered to contribute significantly to IE if the p value was below 0.05. The total amount of variance in IE explained by the models was expressed with the R^2^. IBM SPSS statistics version 22 was used for all analyses.

## Results

**Fig. 1:**
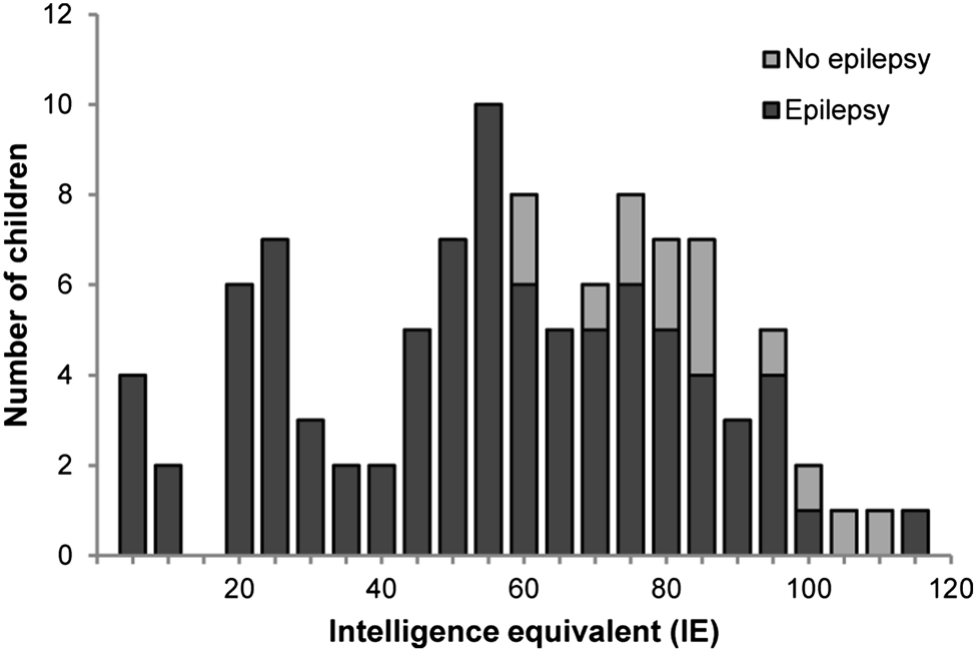
Intelligence equivalent (IE) measured by standard cognitive tests (no epilepsy *n* = 14, epilepsy *n* = 88)

Characteristics of 102 children included in this study are shown in Table 1. IE was measured at a median age of 8.2 years. The median IE of all children was 59 (IQR 39–78) and the median IE of children with epilepsy was 55 (IQR 30–73). IE values are shown in Fig. 1, for children with epilepsy and children without epilepsy. Distribution of IE values of the total study population was shifted towards lower values compared to values in the general population. Median IE in children with epilepsy was 55, and in children without epilepsy 81. Children with epilepsy had lower IE values (*p* < 0.001).

**Table 1:** Description of data from all children (*n* = 102), and the subgroup of children with epilepsy (*n* = 88)

Variable	All children (*n* = 102), number (%)/median (IQR)	Children with epilepsy (*n* = 88), number (%)/median (IQR)
Gender male	53 (52)	46 (52)
Age at cognitive testing (years)	8.2 (4.7–12.0)	8.0 (4.0–11.9)
Intelligence equivalent (IE)	59 (39–78)	55 (30–73)
Mutation		
TSC1	27 (27)	21 (24)
TSC2	67 (66)	60 (68)
NMI	6 (6)	5 (6)
Epilepsy	88 (86)	88 (100)
Infantile spasms	37 (36)	37 (42)
Age at seizure onset (months)	8 (4–24)	6 (3–16)
Number of AEDs used	1 (0–3)	2 (1–4)
Success of first AED	75 (74)	61 (70)
Vigabatrin used		
First AED	33 (32)	19 (22)
Second AED or later	30 (29)	30 (34)
Never used	39 (38)	39 (44)
Corticosteroid treatment	5 (5)	5 (6)
Age of walking independently (months)	18 (14–22)	18 (14–23)

*IQR* inter quartile range, *NMI* no mutation identified, *AED* anti-epileptic drug

Variables included in our regression models and their effect on IE are shown in Table 2 for all included children and in Table 3 for the subgroup of children with epilepsy. Univariable regression analysis of all children showed lower IE values if the child had epilepsy, if the child had infantile spasms, when a larger number of AEDs was used, if vigabatrin was used as first or second drug, if corticosteroid treatment was used, and if a child was older when it could walk independently. IE values were higher when seizure onset was at a later age, and when treatment success was achieved by the first AED. No significant effect of mutation in TSC1 or TSC2 was found.

**Table 2:** Analysis results of all children (*n* = 102)

Variable	Univariable *β*	95% CI	*p* value	Multivariable *β*	95% CI	*p* value
Mutation						
TSC1	17.7	−2.0 to 37.4	0.078	5.7	−12.3 to 23.7	0.534
TSC2	−3.3	−21.6 to 15.1	0.728	−3.5	−20.3 to 13.2	0.679
Epilepsy	−28.4	−42.3 to −14.6	0.000	−8.8	−28.5 to 10.9	0.383
Infantile spasms	−18.3	−28.4 to −8.2	0.000	2.0	−10.0 to 13.9	0.746
Age at seizure onset (months)	1.7	1.3 to 2.2	0.000	1.2	0.4 to 2.0	0.005
Number of AEDs used	−6.3	−8.6 to −4.0	0.000	−0.9	−4.7 to 2.9	0.634
Success of first AED	15.7	4.0 to 27.4	0.008	−0.2	−12.3 to 11.8	0.972
Vigabatrin used						
First AED	−0.9	−12.9 to 11.1	0.881	−3.3	−17.7 to 11.1	0.654
Second AED or later	−14.6	−26.9 to −2.3	0.020	−4.5	−17.8 to 8.8	0.509
Never used	*x*	*x*	*x*	*x*	*x*	*x*
Corticosteroid treatment	−33.2	−56.1 to −10.3	0.005	−11.8	−34.9 to 11.3	0.318
Age of walking independently (months)	−2.1	−3.2 to −0.9	0.000	−0.3	−1.5 to 0.9	0.622

*CI* confidence interval, *AED* anti-epileptic drug

**Table 3:** Analysis results of children with epilepsy (*n* = 88)

Variable	Univariable *β*	95% CI	*p* value	Multivariable *β*	95% CI	*p* value
Mutation						
TSC1	16.6	−4.4 to 37.5	0.121	4.1	−16.2 to 24.4	0.691
TSC2	−4.2	−23.4 to 15.0	0.666	−6.1	−24.8 to 12.6	0.523
Infantile spasms	−13.4	−24.0 to −2.8	0.013	1.7	−10.9 to 14.2	0.796
Age at seizure onset (months)	1.6	1.1 to 2.2	0.000	1.2	0.3 to 2.0	0.007
Number of AEDs used	−5.3	−7.8 to −2.8	0.000	−0.9	−4.8 to 3.1	0.661
Succes first AED	11.1	−0.8 to 23.1	0.068	0.5	−12.2 to 13.2	0.937
Vigabatrin used						
First AED	−16.3	−29.9 to −2.6	0.019	−3.2	−18.3 to 11.9	0.675
Second AED or later	−14.6	−26.4 to −2.7	0.016	−4.9	−18.9 to 9.0	0.491
Never used	*x*	*x*	*x*	*x*	*x*	*x*
Corticosteroid treatment	−29.3	−51.9 to −6.8	0.011	−11.8	−36.0 to 12.4	0.340
Age of walking independently (months)	−2.0	−3.2 to −0.8	0.001	−0.2	−1.6 to 1.3	0.828

*CI* confidence interval *AED* anti-epileptic drug

In a multivariable regression analysis including all variables listed in Table 2, a later age at seizure onset was the only variable that had a significant effect on IE. A delay of seizure onset of 12 months would mean an increase in IE of 14.0 points.

To assess predictive effects in children with epilepsy only, analyses were repeated excluding all children without epilepsy. Analysis of 88 children with epilepsy showed lower IE values if the child had infantile spasms, when a larger number of AEDs was used, if vigabatrin was used as first or second drug, if corticosteroid treatment was used, and if a child was older when it could walk independently. IE values were higher when seizure onset was at a later age. No correlation was found between IE and mutation in TSC1 or TSC2. The multivariable analysis showed that only a later age at seizure onset was independently predictive of IE. A delay of seizure onset of 12 months would mean an IE increase of 14.2 points in these children.

**Fig. 2:**
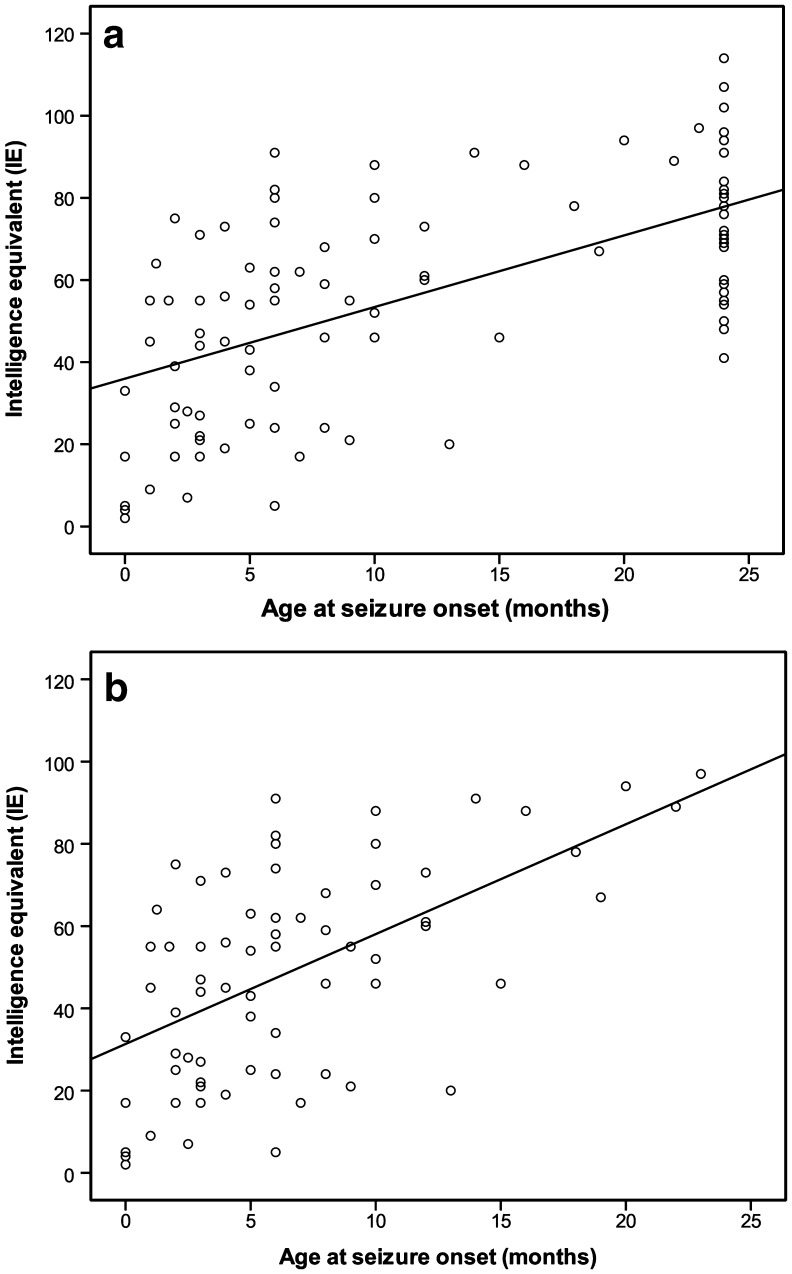
Correlation of intelligence equivalent (IE) with age at seizure onset. **a** Correlation including all children (*n* = 102). 37% of the variance in IE is explained by age at seizure onset. Children without epilepsy are set at 24, children in whom epilepsy started after 24 months are also set at 24. **b** Children in whom epilepsy started within 24 months of age (*n* = 72). 32% of the variance in IE is explained by age at seizure onset

The correlation of IE with the age at seizure onset is shown in Fig. 2. Figure 2a shows the correlation with IE for all children, in whom 28% of IE variation is explained by the age at seizure onset. Figure 2b shows the correlation of IE variation with the age at seizure onset in children who had their first seizure within the first 24 months of life. 32% of IE variation is explained by age at seizure onset in this subgroup.

## Discussion

In this study we used a multivariable regression model to confirm factors that were previously reported to contribute to IE, and to identify new factors contributing to IE. We specifically focused on the possible determinants in the first 24 months of life. We found that age at seizure onset could independently predict IE in our cohort of 102 TSC patients. Our analysis showed a 1.2 points increase of IE for every month the child does not develop epilepsy. This is an IE increase of 14 points for every year the child remains seizure free. This is a clinically relevant increase, as it could mean the difference between being dependent on other individuals throughout life, or going to school and leading an independent life. The variation in IE is explained for 28% by age at seizure onset in the total population, and for 32% in a subpopulation of children with epilepsy. Other factors, including details on the treatment of epilepsy, were found to be predictors of cognitive development in a univariable analysis, but had no significant effect after correcting for the age at seizure onset. Therefore, we could not make a prediction model for cognitive development in children with TSC.

Epilepsy is known to negatively influence cognitive functioning [9]. The age at seizure onset has also been previously correlated with cognition, and patients suffering from epilepsy starting in infancy are at high risk of reduced cognitive abilities. This has also been shown for patients with TSC [1, 7]. Our study shows that, of all the factors included in our multivariable statistical model, only the age at seizure onset was an independent predictor of cognitive development.

Most studies correlating cognitive functioning with patient-related factors have only used univariable analysis methods. Our study confirms that most factors related to epilepsy treatment, for example the number of AEDs and corticosteroid treatment, are correlated to cognitive function when analyzed separately. If they are corrected for the age at seizure onset; however, this effect disappears. This underscores the importance of multivariable analyses, in which any association is corrected for possible confounding factors. Our model confirms previous findings from Jansen et al. [7], showing that factors related to cognitive development are highly intertwined, and we should be cautious about giving parents information on the development of their child based on studies using univariable analyses.

Even though studies suggest that early and effective treatment of epilepsy is beneficial for cognitive development [5], the lack of direct significant correlations found in our analyses is not a new finding. Previous studies in patients with TSC have also struggled to show better cognitive development with successful epilepsy treatment, when trying to find variables independent of other factors [15]. This might indicate that the response to epilepsy treatment is dependent on intrinsic factors of the patient, including the age at seizure onset, and is therefore not an independent predictor. It is also possible that we did not use the right epilepsy treatment variables. For our study, we explored the literature and found that most studies used response to AEDs and AED resistance as variables to correlate with IE. We attempted to make these variables more objective, by including the number of AEDs used, whether the child used corticosteroids or adrenocorticotropic hormone (ACTH), whether the first AED was successful (a seizure frequency decrease of at least 50%), and whether the child used vigabatrin as a first AED, later during treatment, or never. Furthermore, we explored the variable ‘time between the first seizure and the first treatment’, as it has been suggested that a treatment delay might cause a decrease of cognitive capabilities [5]. However, in our cohort, a treatment delay was mostly present in patients with a single seizure without an epileptic encephalopathy on EEG. Treatment delay was, therefore, not a marker of duration of uncontrolled epilepsy, and an AED was usually started when a second seizure occurred. Therefore, a long period between first and second seizure might in our cohort be a marker for a good prognosis. A delay in treatment of infantile spasms may still be an independent prognostic factor. However, due to the small number of children with infantile spasms in our study population, we could not analyze this group separately.

Besides factors related to epilepsy and epilepsy treatment, several other factors have also been correlated with cognitive functioning, including if the patient had a mutation in *TSC1* or *TSC2* [14]. A recent study made a new classification of mutations based on the length of the amino acid tail at the C-terminal in patients with a frameshift or nonsense mutation. A longer C-terminal was correlated with a decreased cognitive functioning in patients with a *TSC1* mutation, and increased cognitive function in patients with a *TSC2* mutation [16]. In contrast to these findings, in a univariable analysis of our cohort, *TSC1* mutations leaving a longer C-terminal tail seemed to correlate with better cognitive functioning. For *TSC2* mutations we could not find a relationship with the length of the C-terminal tail. However, the number of patients with a frameshift or nonsense mutation in our cohort was only 32. Larger cohorts may shed light on the relation between the C-terminal tail and cognitive functioning.

Another factor that has been previously correlated with cognitive function is lesion burden on brain MRI, including the number of cortical tubers, the proportion of brain occupied by tubers and the integrity of white matter [11, 13]. We recognize that this lesion burden plays an important role in brain function, and is therefore likely important in the cognitive development of children with TSC. Unfortunately we did not have MRI data of all children in our study cohort, and could, therefore, not include data on lesion burden in our statistical analysis. Several ongoing studies are collecting data on epilepsy and MRI, including the EPISTOP study (clinicaltrials.gov NCT02098759). This study aims to find biomarkers for epileptogenesis, and will investigate the effect of treatment on epilepsy and cognitive functioning before seizures arise. Combining data from these studies allows the generation of a more detailed predictive model.

We have chosen to use data on possible determinants in the first 24 months of life, since we intended to search for factors in early life that could be correlated with IE. These could aid physicians in making a cautious prediction about the impact of epilepsy and the cognitive outcome of a child. Cognitive development in patients with TSC is very difficult to predict. It would be useful for parents to have an indication of the future abilities of their child, to find the appropriate support and schooling for their child. Finding predictors can also help select children at risk for cognitive problems for future intervention trials.

Our study had several limitations. The children in our study were all treated at a university hospital, which may have introduced a bias towards children who were more severely affected by TSC. Furthermore, some children were treated before the current treatment guidelines for TSC related epilepsy were introduced [3, 10], which might have led to treatment regimens that were not up to the current treatment standard. In addition, our analyses were not able to distinguish between correlation and causality. The question remains whether an early age at seizure onset causes decreased cognitive functioning, or whether the two are caused by the same intrinsic mechanism.

In conclusion, our study shows that, in our study population of 102 children with TSC, age at seizure onset is the only factor that independently predicts cognitive functioning later in life. Finding appropriate markers of early development that can help to predict the cognitive outcome of children with TSC could aid parents and physicians in finding the appropriate support and schooling for these patients, to optimize their chances and outcome in life.

## Electronic supplementary material

Below is the link to the electronic supplementary material.

Supplementary material 1 (DOCX 97 kb)

## References

[1] Bolton PF, Clifford M, Tye C, Maclean C, Humphrey A, le Marechal K, Higgins JNP, Neville BGR, Rijsdjik F, Yates JRW, Grp TSS (2015). Intellectual abilities in tuberous sclerosis complex: risk factors and correlates from the Tuberous Sclerosis 2000 Study. Psychol Med.

[2] Chu-Shore CJ, Major P, Camposano S, Muzykewicz D, Thiele EA (2010). The natural history of epilepsy in tuberous sclerosis complex. Epilepsia.

[3] Curatolo P, Jozwiak S, Nabbout R, Meeting PTC (2012). Management of epilepsy associated with tuberous sclerosis complex (TSC): clinical recommendations. Eur J Paediatr Neuro.

[4] Curatolo P, Moavero R, de Vries PJ (2015). Neurological and neuropsychiatric aspects of tuberous sclerosis complex. Lancet Neurol.

[5] Cusmai R, Moavero R, Bombardieri R, Vigevano F, Curatolo P (2011). Long-term neurological outcome in children with early-onset epilepsy associated with tuberous sclerosis. Epilepsy Behav.

[6] Jansen FE, Braams O, Vincken KL, Algra A, Anbeek P, Jennekens-Schinkel A, Halley D, Zonnenberg BA, van den Ouweland A, van Huffelen AC, van Nieuwenhuizen O, Nellist M (2008). Overlapping neurologic and cognitive phenotypes in patients with *TSC1* or *TSC2* mutations. Neurology.

[7] Jansen FE, Vincken KL, Algra A, Anbeek P, Braams O, Nellist M, Zonnenberg BA, Jennekens-Schinkel A, van den Ouweland A, Halley D, van Huffelen AC, van Nieuwenhuizen O (2008). Cognitive impairment in tuberous sclerosis complex is a multifactorial condition. Neurology.

[8] Joinson C, O’Callaghan FJ, Osborne JP, Martyn C, Harris T, Bolton PF (2003). Learning disability and epilepsy in an epidemiological sample of individuals with tuberous sclerosis complex. Psychol Med.

[9] Kellermann TS, Bonilha L, Lin JJ, Hermann BP (2015). Mapping the landscape of cognitive development in children with epilepsy. Cortex.

[10] Krueger DA, Northrup H, Complex ITS (2013). Tuberous Sclerosis Complex Surveillance and Management: Recommendations of the 2012 International Tuberous Sclerosis Complex Consensus Conference. Pediatr Neurol.

[11] O’Callaghan FJK, Shiell IW, Osborne JP, Martyn CN (1998). Prevalence of tuberous sclerosis estimated by capture-recapture analysis. Lancet.

[12] Overwater IE, Bindels-de Heus K, Rietman AB, ten Hoopen LW, Vergouwe Y, Moll HA, de Wit MCY (2015). Epilepsy in children with tuberous sclerosis complex: chance of remission and response to antiepileptic drugs. Epilepsia.

[13] Raznahan A, Higgins NP, Griffiths PD, Humphrey A, Yates JRW, Bolton PF (2007). Biological markers of intellectual disability in tuberous sclerosis. Psychol Med.

[14] van Eeghen AM, Chu-Shore CJ, Pulsifer MB, Camposano SE, Thiele EA (2012). Cognitive and adaptive development of patients with tuberous sclerosis complex: a retrospective, longitudinal investigation. Epilepsy Behav.

[15] Winterkorn EB, Pulsifer MB, Thiele EA (2007). Cognitive prognosis of patients with tuberous sclerosis complex. Neurology.

[16] Wong HT, McCartney DL, Lewis JC, Sampson JR, Howe CJ, de Vries PJ (2015). Intellectual ability in tuberous sclerosis complex correlates with predicted effects of mutations on TSC1 and TSC2 proteins. J Med Genet.

